# Intra-familial dynamics of mental distress during the Covid-19 lockdown

**DOI:** 10.1038/s41398-026-03876-z

**Published:** 2026-02-17

**Authors:** Johanne H. Pettersen, Espen Eilertsen, Laura Hegemann, Laurie J. Hannigan, Ingunn Olea Lund, Pia M. Johannessen, Elizabeth C. Corfield, Eivind Ystrom, Ole A. Andreassen, Alexandra Havdahl, Ragnhild E. Brandlistuen, Helga Ask

**Affiliations:** 1 PsychGen Center for Genetic Epidemiology and Mental Health, Norwegian Institute of Public Health, Oslo, Norway; 2 Department of Psychology, University of Oslo, Oslo, Norway; 3 Psychiatric Genetic Epidemiology Group, Research Department, Lovisenberg Diaconal Hospital, Oslo, Norway; 4 Population Health Sciences, Bristol Medical School, University of Bristol, Bristol, UK; 5 Department of Child Health and Development, Norwegian Institute of Public Health, Oslo, Norway; 6 PROMENTA Research Center, Department of Psychology, University of Oslo, Oslo, Norway; 7 Centre for Research on Equality in Education, Faculty of Educational Sciences, University of Oslo, Oslo, Norway; 8 Center for Precision Psychiatry, Division of Mental Health and Addiction, Oslo University Hospital, Oslo, Norway; 9 Institute of Clinical Medicine, University of Oslo, Oslo, Norway; 10 Division of Mental and Physical Health, Norwegian Institute of Public Health, Oslo, Norway; 11 Department of Children and Families, Norwegian Institute of Public Health, Oslo, Norway; 12 The Norwegian Mother, Father, and Child Cohort Study (MoBa), Norwegian Institute of Public Health, Oslo, Norway

**Keywords:** Human behaviour, Comparative genomics

## Abstract

Lockdowns and social restrictions imposed in response to the Covid-19 pandemic intensified the proximity and reciprocal exposure among members of nuclear families. It is unclear how variation in mental distress during this period is attributed to potential influences of family members. This study used genetic data from adolescents (n = 4 388), mothers (n = 27 852) and fathers (n = 25 953), to disentangle the contributions of parent-driven, child-driven, and partner-driven components to mental distress during the first two months of the Covid-19 lockdown. Separate models also included adolescents’ non-pandemic mental distress as outcomes (n = 13 484). Trio genome-wide complex trait analyses separated two types of genetic components; *direct*–how an individual’s genotype is associated with their own mental distress, and *indirect*–how an individual’s genotype is associated with the mental distress of family members. A trio polygenic score (PGS) design was used to investigate associations of specific genetic liability factors with mental distress, and whether these changed over time (PGS×time). Results suggest that family-level genetic factors contribute to mental distress; variance components capturing indirect genetic effects accounted for 10% of adolescent mental distress (mother-driven), 2–3% of maternal (partner-driven), and 5% of paternal mental distress (child-driven). Mothers’ depression and ADHD PGS were positively associated with fathers’ mental distress. No PGS×time interactions were found. Direct genetic effects accounted for 9–10% variance in mental distress across family members, partly explained by genetic variants associated with anxiety, depression, ADHD and neuroticism. These findings highlight the importance of family dynamics and emphasize the potential value of including family members in mental health interventions.

## Introduction

During the first weeks of the Covid-19 pandemic, many families were confined at home together. This abrupt environmental disruption, which for many also saw increased stress, uncertainty and mental distress [1, 2], provides a unique opportunity to examine the family dynamics of mental health.

Previous studies have suggested that family dynamics play a role in individuals’ mental distress (i.e., symptoms of anxiety and depression), even after adjusting for the genetic and environmental factors that are shared within families. The majority of this literature emphasizes what we can call *parent-driven effects*: how the role and behavior of parents shape the mental health of children, through for example parenting [3] or maternal lifestyle during pregnancy [4, 5]. Recently, such parental influences have been investigated using polygenic scores (PGS) related to specific traits (e.g., ADHD and depression). Parental PGS are included as exposures and offspring mental health as outcome in regression models. By adjusting such models for the child’s own PGS (the transmitted genetic variants), we can estimate possible parent-driven effects on the child’s outcome. Such indirect genetic effects have been identified for common genetic variants associated with autism, ADHD, and educational attainment on child neurodevelopmental traits [6], but with less evidence for other outcomes (e.g., on conduct problems [7], and of the educational attainment PGS on children’s depressive, anxiety, or ADHD traits [8]).

Some literature also focuses on how romantic partners influence each other, through *partner-driven effects*. Although partner similarity across a range of traits is evident from the initial stages of a relationship (arising through processes like social homogamy and assortative mating), convergence over time has also been observed [9, 10]. Partners likely influence each other’s mental health through their behavior and interactions. For example, relationship conflict and poor communication have been associated with higher maternal depression risk postpartum [11].

Fewer studies investigate *child-driven effects*, how children may influence the mental health of their parents. For instance, adoptions studies have shown that child emotional and behavioral problems are associated with mental health outcomes in adoptive parents [12, 13]. In one study on the association between offspring sleep problems and maternal depression, child-driven effects appeared stronger than the mother-driven [14]. Child-driven effects have also been investigated using the trio-PGS design. For example, a recent study found evidence for both child- and partner-driven effects on maternal depression, through PGS for a general psychopathology factor, varying in strength across life stages [15].

In addition to the search for specific traits or behaviors in one family member that are associated with mental health outcomes in another (trait-based approaches), variance-based approaches [16] offer an alternative framework for investigating parent-, partner- and child-driven components. In these approaches, specific variables related to traits or behaviors are not selected, but variance in the outcome of interest is partitioned based on its underlying source, estimated based on patterns of covariance across family members. Classical twin modelling is one example of a variance-based approach, disentangling variance explained by genetic, shared environmental and unique environmental influences. Twin studies have estimated the heritability of anxiety and depression at 30–50% [17, 18], with shared environmental factors accounting for up to 30% of variance in childhood, though this influence typically diminishes in adulthood [19].

Another recently developed variance-based approach is trio genome-wide complex trait analysis (Trio-GCTA) [20], which uses data on genome-wide common genetic variants from mother, father, and offspring trios to separate direct and indirect genetic effects. Providing a broad perspective on the overall genetic contributions within families, the trio-GCTA offers a valuable initial step in understanding family dynamics, which can be complemented by subsequent trait-based approaches. Previous Trio-GCTA studies have estimated that partner- and child-driven components account for between 0–14% variance in maternal depression across several timepoints [21], and that parent-driven components explain 8–16% variance in conduct, inattention and hyperactivity [22] in 8 year-old children. Mother-driven components have also been shown to contribute 6–10% of variance in children’s early neurodevelopmental traits using this method [6].

Most previous literature investigating parent-driven associations has relied on maternally reported child outcomes, potentially biasing the observed effects of mothers on children [6, 22, 23]. Therefore, investigating family dynamics using trio self-reported data is of importance. Also, few studies have examined family dynamics in families with older children ( > 8 years), an area of particular importance given the increased mental distress among adolescents. A recent systematic review described the Covid-19 pandemic as an intensifier of intergenerational risk of mental health problems among children [24].

In this pre-registered study, we used longitudinal and cross-sectional data on mental distress reported by mother, father, and adolescent offspring trios during the first two months of the Covid-19 lockdown in Norway. Using both a variance-based (Trio-GCTA), and a trait-based (Trio-PGS) approach, we aimed to investigate 1) to what extent adolescents’, mothers’ and fathers’ mental distress was influenced by direct and indirect genetic effects 2) to what extent these effects could be explained by genetic variants associated with anxiety, depression, ADHD, neuroticism and anorexia and 3) whether the magnitude of direct and indirect effects changed over time in lockdown.

## Methods

### Study design and sample

The Norwegian Mother, Father, and Child Cohort Study (MoBa) is a population-based pregnancy cohort study conducted by the Norwegian Institute of Public Health [25, 26]. Participants were recruited from all over Norway from 1999–2008. The women consented to participation in 41% of the pregnancies. The parents provided informed consent on behalf of themselves and their children at birth. The cohort includes approximately 114 500 children, 95 200 mothers, and 75 200 fathers. The establishment of MoBa and initial data collection was based on a license from the Norwegian Data Protection Agency and approval from The Regional Committees for Medical and Health Research Ethics. The MoBa cohort is currently regulated by the Norwegian Health Registry Act. The current study was approved by The Regional Committees for Medical and Health Research Ethics (14140). Blood samples were obtained from both parents during pregnancy and from mothers and children (umbilical cord) at birth [27]. Genotyping of MoBa, quality control, phasing, imputation, and post-imputation quality control has previously been described [28]. MoBa data was linked to the Medical Birth Registry (MBRN), a national health registry containing information about all births in Norway.

Since March 2020, parents and 16-18-year-old adolescents in MoBa were invited to respond to biweekly Covid-19 questionnaires. Three of these questionnaires were sent out during the lockdown period in Norway between March and May 2020, including a measure of mental distress. In this study, we leveraged data on complete family trios of 15-18-year-old adolescents (n = 4 388), mothers (n = 27 852), and fathers (n = 25 953) responding to at least one of these questionnaires. Mental distress among adolescents was also assessed in separate questionnaires sent out to 14-16-year-olds (from 2017-2023), resulting in a total sample of 13 484 adolescents. Trio-GCTA samples were restricted to unrelated trios (See flow chart in Supplementary Figure 1). This paper was preregistered in the open science framework (OSF): https://osf.io/2npgt. See Supplementary Table 1 for a complete list of deviations from the preregistration.

### Outcome

Mental distress was measured by the 5-item version of the Hopkin’s Symptom Checklist [29]. Each item was scored on a scale of 1 (not bothered) to 4 (very bothered), based on experiences during the last two weeks. A mean SCL-5 score for each of the three timepoints during the lockdown period was estimated and multiplied by the total number of items. Additionally, an overall SCL-5 mean score was created for all adolescents (including 1–4 measurements). All SCL-5 scores were log-transformed to reduce skewness. Due to limited power in the adolescent sample measured during the pandemic, we created an overall mental distress score for all adolescents who had responded to SCL-5 between 14 and 18 years (between 2017–2023) by estimating the mean across available reports (ranging from one to four measurements). All SCL-5 scores were log-transformed and standardized before being included in the analyses. Respondents with fewer than two items answered did not receive an SCL-5 score.

For supplementary PGS-analyses on adolescents, we included all the available measurements (one to four) for adolescents who had responded to SCL-5 at any time instead of creating a mean score as in the trio-GCTA analyses. For supplementary analyses on pre-pandemic SCL-5, mothers responded to a questionnaire when their child was 8 years old (between 2011–2017), and fathers responded to a questionnaire between 2015–2016.

### Exposures

A PGS quantifies measured genetic liability to a particular condition by summing an individual’s risk alleles, each weighted by their effect size in a previous genome-wide association study (GWAS) [30]. PGS for each family member were generated using the LDpred2 software [31], a Bayesian-based method, based on an established pipeline [32]. This pipeline subsets the genetic dataset to HapMap3 [33] variants (common SNPs that have been identified across populations) and uses linkage disequilibrium (LD) panels created based on existing LD matrices from UK Biobank. The “LDPred2-auto” option was selected for the PGS scores. To prevent overlap, MoBa participants have been excluded from summary statistics for each trait. PGS were subsequently adjusted for the first 20 genomic principal components, genotype, and imputation batch. PGS were created for anxiety [34], depression [35], neuroticism [36], ADHD [37], and anorexia nervosa (AN) [38].

Confounding variables included: adolescents’ sex assigned at birth (Male/Female), child age (in 2020, range 11-21-years), mothers age (30–63 years), and fathers age (31–75 years). Additionally, a continuous variable for time was created, representing the number of days between March 12^th^, 2020, and the completion of a given questionnaire. A variable on household status was created based on registered household status in 2019 from Statistics Norway. Family member trios (mother, father and child) registered in the same household were labeled “living together”. Family members not living together were registered as missing. This variable was used to limit the dataset in sensitivity analyses to complete family trios registered as living in the same household. In this paper, we use the term “effects” to refer to model-estimated parameters, which do not imply causality.

### Statistical analyses

#### Variance-based approach

To disentangle the direct and indirect genetic variance components on mental distress in family trios, we used Trio-GCTA. This method uses a genomic relatedness matrix (GRM) based on common genome-wide SNPs from the individuals in the trio. The covariances between direct and indirect genetic components can be interpreted as gene-environment correlations [20]. The Trio-GCTA method allows us to estimate unconfounded genetic associations of each family members on adolescent’s, mother’s and father’s mental distress. The variance components estimated in the model can be seen in Eq. 1.

1 $$Var({\Upsilon })={\sigma }_{m}^{2}+{\sigma }_{p}^{2}+{\sigma }_{o}^{2}+{\sigma }_{om}+{\sigma }_{op}+{\sigma }_{e}^{2}$$

When the adolescent is the focal individual, the *σ*^*2*^_*o*_ is interpreted as the direct genetic effect, and the *σ*^*2*^_*m*_ and *σ*^*2*^_*p*_ are the variance attributed to the maternal and paternal indirect effects, respectively. The *σ*_*om*_ represents the covariances between the child and maternal indirect effects, and *σ*_*op*_ are the covariances between the child’s direct effect and paternal indirect effects. *σ*^*2*^_*e*_ represents the residual variance not explained by the genetic effects. Covariance between maternal and paternal indirect genetic effects *σ*_*mp*_ is estimated but we do not expect it to contribute to the total variance of the phenotype.

Five models were run for each focal individual (see Supplementary Table 2): 1) A full model including all parameters; 2–3) Two models including the direct effect of the focal individual and the indirect effect of each of the other family members (in separate models) in addition the covariance between the direct and indirect effects. For instance, when the adolescent is the focal individual, one model included the child’s direct effect, the maternal indirect effect and the covariance between maternal and child effects, and an error term (Model MO), while the second model substituted the maternal with the paternal indirect effect (Model FO); 4) A model with only direct effects (and error term); and 5) a null model including only the error term.

**Fig. 1:**
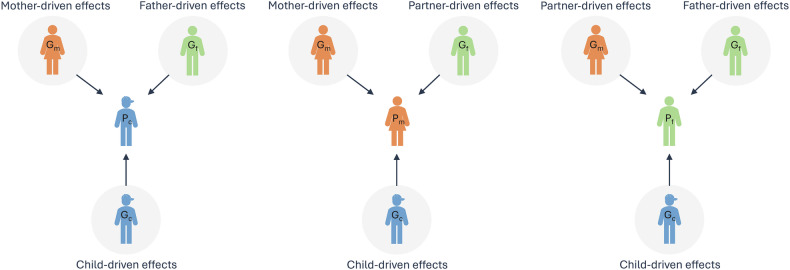
Illustrative model of mother-, father-, partner-, and child-driven effects on mental distress in family trios. As the focal individual changes (mental distress in mother, father or adolescent), the interpretation of the effects changes. When the adolescent is the focal individual, the child-driven effects represent direct effects and mother- and father-driven effects are indirect genetic effects. When the mother/father is the focal individual the child- and partner-driven effects are the indirect effects and the mother/father-driven effects represent the direct genetic effects.

For adolescents, due to limited power in the Covid-19 sample, one model was run including the overall SCL as outcome. For mothers and fathers, three separate models were run, one for each SCL measure during the Covid-19 lockdown. As the focal individual changes (adolescent, mother, or father), the interpretation of the model parameters also changes, as can be seen in the illustration in Fig. 1.

Using the Julia programming language [39], and the VCModels.jl package [40] we compared five models for each focal individual (adolescent, mother, father) and timepoint (see eTable 2 for variance components included in the different models). Each model included the fixed effects of child sex, genotype batch, and first 10 principal components.

The best-fitting model was selected based on the lowest Akaike’s Information Criteria (AIC) [41] value. We also reported Bayesian Information Criteria (BIC) and performed Likelihood ratio tests by comparing each model to the full model, estimating whether the nested models provided a significantly worse fit to the data (α < 0.05). However, due to the use of family data and complex models, BIC and likelihood ratio tests might perform worse in selecting the model closest to the true model [42, 43]. We therefore rely on AIC in model selection.

#### Trait-based approach

Multilevel regression models, including the trio PGS as exposures and repeated measures of mental distress as outcome, were conducted in R [44] using the lme4 package [45]. Separate models were run for each focal individual, and for each PGS (i.e., anxiety, depression, ADHD, neuroticism, and AN), 15 models in total. In each model, the PGSs of the mother, father and child were simultaneously added, allowing the regression coefficient for each family member’s PGS to be adjusted for the PGS of the others. The coefficient (β) of the focal individual’s PGS on their own mental distress was interpreted as the direct genetic effect, while the other coefficients of the other family members’ PGSs were interpreted as consistent with indirect genetic effects, adjusted for the focal individual’s PGS). To investigate whether the magnitude of genetic effects changed over time in lockdown, all models were rerun including interaction terms between each PGS and time.

All models were adjusted for time, age of the focal individual, and child’s sex. Random intercepts of the focal individuals ID were added to account for repeated measures. In the models with the adolescent’s outcome, we added a random intercept of maternal ID, to account for siblings. Full information maximum likelihood (FIML) was used to handle missing data. We used false discovery rate (FDR) to adjust for multiple testing for 5 tests (5 PGS). Supplementary trio-PGS analyses were conducted including all adolescents with available SCL-5 data, and sensitivity trio-PGS analyses were run on restricted samples of family trios registered in the same household. In addition, to contextualize the results related to the Covid-19 lockdown, we conducted supplementary analyses running trio-GCTA and trio-PGS models on a subsample of mothers and fathers with pre-pandemic measures of mental distress included as outcomes.

## Results

All participants reported highest level of mental distress at the first timepoint during lockdown (M_adolescents_=1.55,SD = 0.60; M_mothers_ = 1.46,SD = 0.49; M_fathers_ = 1.35,SD = 0.44) and lowest at the third timepoint (M_adolescents_=1.52,SD = 0.59; M_mothers_ = 1.37,SD = 0.46;M_fathers_ = 1.31,SD = 0.44). Phenotypic correlations between family members’ mental distress were small but consistent across timepoints (r ranged between 0.09 to 0.14), and variances indicated modest changes in symptom variability across time (Supplementary Table 3). Sociodemographic characteristics across samples can be found in Table 1.

**Table 1:** Sociodemographic characteristics.

Sociodemographic characteristics	Adolescent trios		Mother trios			Father trios		
	Overall sample (N = 13,484)	Lockdown sample (N = 4,388)	T1 (N = 25,575)	T2 (N = 25,108)	T3 (N = 23,591)	T1 (N = 23,630)	T2 (N = 22,285)	T3 (N = 20,349)
**Observations**	20,162	9,674						
**Child’s sex**								
Male	6,211 (46.1%)	1,878 (42.8%)	12,971 (50.7%)	12,732 (50.7%)	11,955 (50.7%)	12,046 (51.0%)	11,374 (51.0%)	10,344 (50.8%)
Female	7,273 (53.9%)	2,510 (57.2%)	12,608 (49.3%)	12,376 (49.3%)	11,636 (49.3%)	11,584 (40.0%)	10,911 (49.0%)	10,005 (49.2%)
**Mean (SD) age – Children**	16.0 (1.1)	16.9 (0.6)	14.4 (2.0)	14.4 (2.0)	14.4 (2.0)	14.3 (1.9)	14.3 (2.0)	14.3 (2.0)
**Mean (SD) age – Mothers**	46.3 (4.4)	47.1 (4.5)	45.1 (4.8)	45.2 (4.8)	45.2 (4.8)	45.0 (4.8)	45.1 (4.7)	45.2 (4.7)
**Mean (SD) age – Fathers**	48.6 (5.1)	49.4 (5.1)	47.4 (5.5)	47.5 (5.5)	47.5 (5.5)	47.4 (5.4)	47.5 (5.4)	47.6 (5.4)
**Mother’s education**								
Compulsory	389 (2.9%)	183 (4.2%)	1,019 (4.0%)	1,010 (4.0%)	880 (3.7%)	938 (4.0%)	846 (3.8%)	746 (3.7%)
Upper secondary	2,712 (20.1%)	1,144 (26.1%)	5,779 (22.6%)	5,689 (22.7%)	5,195 (22.0%)	5,181 (21.9%)	4,787 (21.5%)	4,299 (21.1%)
Bachelor’s degree	7,368 (54.6%)	2,271 (51.2%)	13,446 (52.6%)	13,228 (52.7%)	12,534 (53.1%)	12,332 (52.2%)	11,677 (52.4%)	10,691 (52.5%)
Master’s degree or Ph.D.	3,008 (22.3%)	788 (18.0%)	5,314 (20.8%)	5,165 (20.6%)	4,967 (21.1%)	5,158 (21.8%)	4,961 (22.3%)	4,605 (22.6%)
**Father’s education**								
Compulsory	767 (5.7%)	289 (6.6%)	1,712 (6.7%)	1,674 (6.7%)	1,518 (6.4%)	1,358 (5.7%)	1,234 (5.5%)	1,078 (5.3%)
Upper secondary	5,123 (20.1%)	1,891 (43.1%)	10,272 (40.2%)	10,118 (40.3%)	9,352 (39.6%)	8,951 (37.9%)	8,259 (37.1%)	7,472 (36.7%)
Bachelor’s degree	4,598 (34.1%)	1,383 (31.5%)	8,385 (32.8%)	8,263 (32.9%)	7,861 (33.3%)	8,160 (34.5%)	7,801 (35.0%)	7,180 (35.3%)
Master’s degree or Ph.D.	2,980 (22.1%)	823 (18.8%)	5,157 (20.2%)	5,005 (19.9%)	4,816 (20.4%)	5,132 (21.7%)	4,968 (22.3%)	4,598 (22.6%)
**Mean (SD) scl-5**	1.56 (0.62)	1.54 (0.59)	1.46 (0.49)	1.42 (0.47)	1.37 (0.46)	1.35 (0.44)	1.34 (0.45)	1.31 (0.44)
**SCL-5 cut off (2.0)**								
No	15,346 (76.1%)	7,411 (76.6%)	21,106 (82.5%)	21,003 (83.7%)	20,139 (85.4%)	20,674 (87.5%)	19,295 (86.6%)	17,720 (87.1%)
Yes	4,818 (23.9%)	2,265 (23.4%)	4,469 (17.5%)	4,105 (16.3%)	3,452 (14.6%)	2,956 (12.5%)	2,990 (13.4%)	2,629 (12.9%)

Mother’s and father’s education is measured as the highest completed level of education in 2020. SD = standard deviation, T1 = timepoint 1, T2 = timepoint 2, T3 = timepoint 3.

*SCL-5*, Hopkins Symptom Checklist (5-item version).

### Trio-GCTA

#### Parental models

For mothers, the best-fitting model at the first timepoint included only direct genetic effects, explaining 9.9% variance in mental distress. For the second and third timepoints, models including direct and father indirect effects revealed the best fit, with father indirect effects contributing 2.9% and 2.6% and direct effects explaining 9.5% and 9.3% of the variance, respectively (Supplementary Table 4).

**Fig. 2:**
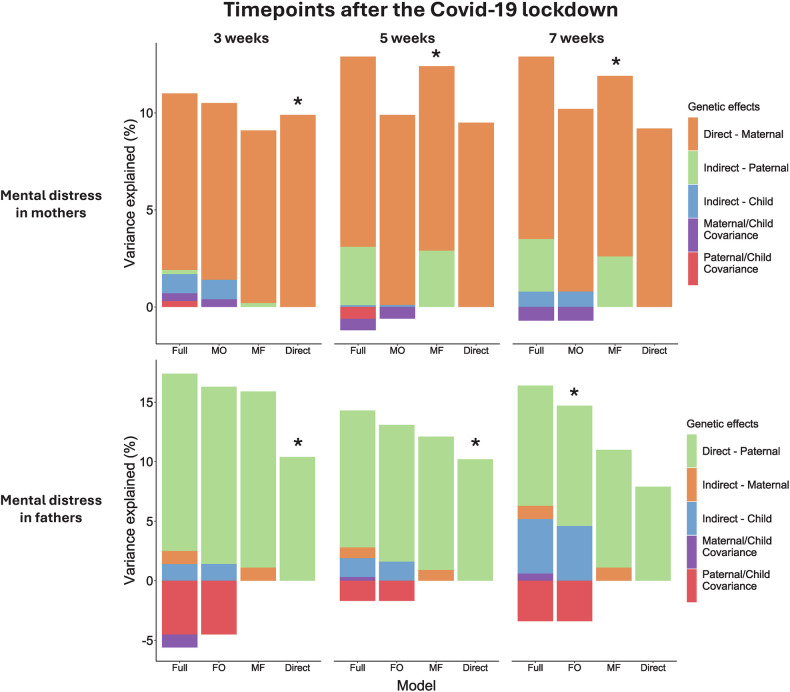
Trio-GCTA model run on mothers’ and fathers’ mental distress measured at three timepoints during the Covid-19 lockdown. The trio-GCTA model on maternal and paternal mental distress. Variance component estimates are presented for each model tested: full model with all parameters included (Full); maternal/paternal direct and child indirect effects (MO/FO); and direct effect model including maternal/paternal genetic effect (Direct). The best-fitting models based on the lowest AIC is indicated by *. Covariance between maternal and paternal effects is estimated in the model but not shown in the figure as we do not expect it to contribute to the variance in maternal/paternal mental distress.

For fathers, the best-fitting model at timepoint 1-2 included only direct effects, explaining 10.4% and 10.2% variance, respectively. At timepoint 3, the optimal model included direct and child indirect genetic effects, explaining 4.6% and 10.1%, respectively, with a negative covariance between these (correlation of −0.49). Figure 2 shows variance explained in each model.

#### Adolescent models

**Fig. 3:**
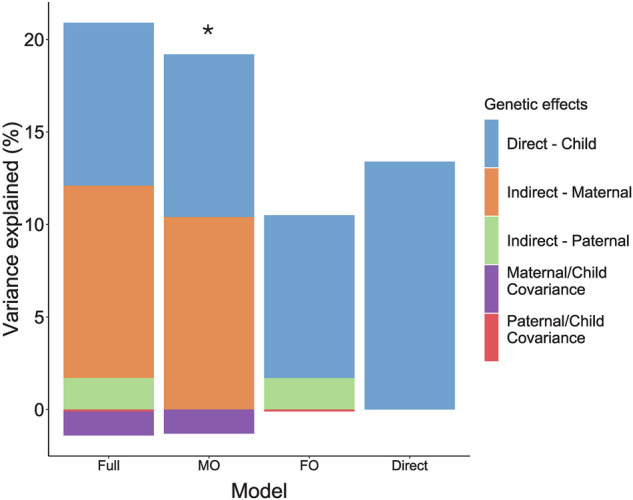
Trio-GCTA model run on adolescents’ mental distress. Variance component estimates are estimated for each model tested: full model with all parameters included (Full model); child direct and mother indirect effects (MO); child direct and father indirect effects (FO); direct effects model only including the child’s genetic effect (Direct). The best-fitting models based on the lowest AIC are indicated by *.

The model estimating direct and maternal indirect genetic effects was best-fitting for adolescent mental distress (Supplementary Table 5), explaining 8.8% and 10.4%, respectively. There was a negative covariance between direct and maternal indirect genetic effects (correlation of −0.13). Figure 3 shows variance explained in each model.

### PGS analyses

The PGS analyses on father’s mental distress revealed indirect genetic effects of mother’s depression_PGS_ (β = 0.02, 95%CI[0.01,0.04]) and ADHD_PGS_ (β = 0.03[0.02,0.04]). There were no significant indirect genetic effects on adolescent or maternal mental distress.

Direct genetic effects were observed across all models for depression_PGS_ (β_adolescent_ = 0.08[0.05,0.12];β_mother_ = 0.11[0.09,0.12];β_father_ = 0.10[0.09,0.12]), and neuroticism_PGS_ (β_adolescent_ = 0.07[0.03,0.11];β_mother_ = 0.11[0.09,0.12];β_father_ = 0.10[0.08,0.11]). We also found direct genetic effects of anxiety_PGS_ (β_mother_ = 0.05[0.03,0.06];β_father_ = 0.05[0.04,0.06]), ADHD_PGS_ (β_mother_ = 0.03[0.01,0.04]; β_father_ = 0.04[0.02,0.05]) and AN_PGS_ (β_mother_ = 0.03[0.02,0.04]; β_father_ = 0.03[0.01,0.04]) on parental mental distress.

**Fig. 4:**
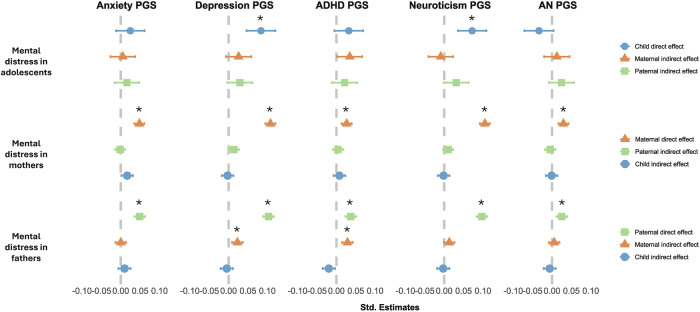
Standardized beta estimates for PGS on adolescents, mothers, and fathers’ mental distress. Blue circles show adolescent genetic effects, maternal genetic effects are indicated by orange triangles, and paternal by green squares. * Indicates significant p-values after FDR correction.

We found no significant interaction effects between PGS and time in lockdown. Standardized estimates can be found in Supplementary Table 6 and Fig. 4.

Supplementary trio-PGS analyses on adolescent overall mental distress (not restricted to pandemic measures), revealed only direct genetic effects (Supplementary Table 7 and Supplementary Figure 2), and sensitivity analyses including only family members registered in the same household revealed similar results (see Supplementary Table 8).

Additional supplementary trio-GCTA and trio-PGS analyses were conducted using pre-pandemic parental mental distress as the outcome. For mothers, the best-fitting trio-GCTA model included both direct genetic effects and indirect effects from partners, explaining 15.2% and 4.2% of the variance in maternal mental distress, respectively (see Supplementary Table 9). The Trio-GCTA models on father’s pre-pandemic mental distress were underpowered, and therefore the null model provided the best fit. Trio-PGS analyses revealed an indirect genetic effect of fathers’ depression PGS on maternal mental distress, however the indirect genetic effect of mothers ADHD PGS on paternal mental distress observed during the pandemic, was not significant in these analyses. Fathers’ direct genetic effects were also not significant for ADHD and AN PGS (see Supplementary Table 10).

## Discussion

In this pre-registered study, we used two complementary approaches to examine within-family processes and mental distress in parents and adolescent offspring during the first Covid-19 lockdown in 2020 in Norway. First, we quantified the total variance in mental distress attributable to indirect and direct genetic components. Next, we investigated to what extent these associations were captured by PGS for anxiety, depression, ADHD, neuroticism, and anorexia nervosa (AN). Last, we investigated whether these effects changed over time. We found evidence consistent with both indirect and direct genetic effects and, while formal comparisons detected no interactions with time spent in lockdown, an increasing proportion of variance was accounted for by indirect genetic components at later waves.

### Mothers are important for adolescent mental distress

In line with previous results based on younger children [23, 46], more than 10% of the variation in adolescent mental distress (measured before and during the pandemic) was linked to mothers’ genes. Previous research has indicated that indirect genetic effects may decline as children mature; for example, a decrease in the influence of shared environments with age has been repeatedly observed in twin studies [19]. The mother-driven associations observed in younger children has been suggested to be partly explained by a maternal rating bias, as the mother often report on the mental health outcomes of younger children. Our results consistent with mother-driven (and lack of father-driven) effects on adolescent *self-reported* mental distress are therefore particularly novel.

Mother-driven effects are supposedly mediated through the mother’s behavior or the family environment she provides. For instance, genetic predisposition to higher neuroticism could affect parenting style, which in turn could impact child mental health. A study on 8-year-old children (in MoBa), suggested that maternal anxiety and depression symptoms partially mediated the indirect maternal effects [23] In our trio-PGS analyses, however, we did not find support for indirect maternal effects for any of the included PGS. On the other hand, the correlation between the child genetic effect and maternal indirect effect (gene-“environment” correlation) was close to zero in the trio-GCTA model. Based on this lack of correlation we would not expect the same PGS to be important for both indirect and direct genetic effects. Hence, the mother-driven associations (observed in the trio-GCTA results) could be explained by traits and conditions not included in our PGS analyses. For example, reflecting environmental influences in utero and during childhood.

### Child- and partner-driven effects on parental mental distress

We found no evidence consistent with indirect genetic effects in parental mental distress at the first timepoint. However, in the following two bi-weekly data collections, father-driven associations accounted for 2–3% of the variance in mothers and child-driven associations explained 5% of variance in fathers’ mental distress at the third timepoint.

Our results build on and extend previous research suggesting that between 0–14% variance in maternal depression is explained by indirect genetic components (including both child- and partner-driven), when measured between the child was 6 months and 8 years old [21]. Uniquely, our study separates the genetic contributions of children and partners.

Our trait-based approach did not support that the included PGS explained the observed father-driven and child-driven effects. However, we did find evidence consistent with indirect genetic effects of maternal depression and ADHD PGS on paternal mental distress. Higher depression or ADHD PGS in mothers was associated with elevated levels of mental distress in fathers. In the full (not the best-fitting) Trio-GCTA model for fathers’ mental distress, indirect maternal effects explain 1% of the variance, supporting that such influences could exist. Interestingly, supplementary pre-pandemic trio-PGS analyses revealed an indirect genetic association of fathers’ depression PGS on maternal pre-pandemic mental distress, while the observed effect of mothers’ ADHD PGS on fathers’ mental distress during the Covid-19 lockdown was not evident before the pandemic. The PGS generally explain very little variance in the outcome, which may indicate that some indirect effects estimated from the PGS are too small to be detected in the larger picture we get from the trio-GCTA models. Although most family-driven associations were not captured by the included PGS, the strength of an indirect PGS effect likely depends on both how strongly the PGS predicts the family member’s own trait (e.g., anxiety or depression) and how strongly that trait is associated phenotypically with the other family member’s mental distress. Trio-GCTA and trio-PGS, while complementary, might yield inconsistent results as they operate at different scales.

### Direct genetic effects

As expected by previous studies [21, 23], direct genetic effects explained 9–10% variation in mental distress across models. These numbers are comparable to previous results from the same cohort. For example, direct effects explained 5–19% of variance in anxiety and depression symptoms at age 8 [23] and 5–14% in maternal depression symptoms [21]. Trait-based analyses suggested that the direct effect could partly be explained by PGS for the included traits, particularly depression and neuroticism. Consistent with previous studies [21], our analyses revealed a negative covariance between the direct and indirect effects on adolescent mental distress, suggesting that the same genetic variants in mothers and their children may have opposing influences on mental distress, highlighting a possible gene-environment correlation [20]. However, this captures only one facet of gene-environment interplay, and other family and environmental factors likely play more immediate roles in shaping mental distress.

### Time in lockdown

Evidence from our study as to whether or not time in lockdown changed the intra-familial dynamics of mental distress was mixed. While we observed a pattern of increasing proportion variance explained on parental mental distress in the trio-GCTA models across time in lockdown, we did not find that the direct and indirect genetic effects changed as a function of time in lockdown in the trio-PGS models. However, most of the family-driven effects were not captured by the included PGS. The pattern of increase in indirect genetic effects seen across time in the trio-GCTA models might reflect other processes than those captured by the PGS. Supplementary trio-GCTA models on maternal pre-pandemic mental distress revealed a similar pattern as the later timepoints during the lockdown, with variance components consistent with both direct and indirect genetic effects, indicating that the initial timepoint during the Covid-19 lockdown could reflect a more extreme situation where external stressors may temporarily outweigh typical family-level genetic effects.

It is uncertain whether the pandemic lockdown shaped the findings on adolescent mental distress. Due to power limitations, we included data on adolescents reporting mental distress outside of the pandemic. Further research on adolescent mental distress is needed to examine associations independently of the pandemic lockdown.

#### Limitations

Our study has some limitations that are relevant to consider when interpreting our results. First, in the trio-GCTA model comparisons, model fit revealed small differences, and the statistical support for the selected best-fitting models might be limited. Second, the MoBa sample consists of higher educated and healthier families [47], and limiting our analyses to complete trios have likely resulted in a highly selected sample. Third, adolescent participation during lockdown was lower than parental participation, due to the limited age-range (16–18 years) and declining response rates across timepoints, which may have introduced some selection bias in adolescent models. Fourth, factors like assortative mating, residual population stratification, and sibling effects are likely to be captured in our estimates of indirect genetic effects, limiting the extent to which they can be interpreted as evidence of exposure-based environmental effects [22]. Finally, our data were based on a quality control restricted to participants of European ancestry, limiting our results’ generalizability.

## Conclusion

Our findings provide evidence consistent with intra-familial processes in shaping mental distress. Mother-driven components contributed to more variance explained than direct genetic effects in adolescent mental distress. Father-driven components contributed to maternal mental distress, and both partner-driven and child-driven components contributed to mental distress in fathers. These findings highlight the complex interplay of genetic and environmental factors in shaping mental health within nuclear families, particularly during periods of acute environmental stressors such as the Covid-19 lockdown. Understanding these dynamics is important in designing mental health interventions.

## Supplementary information

Supplementary Material

## Data Availability

Analysis code is publicly available on GitHub: https://github.com/psychgen/Family-trios-Covid19.
